# Metastases to the Pancreas: Computed Tomography Imaging Spectrum and Clinical Features: A Retrospective Study of 18 Patients With 36 Metastases

**DOI:** 10.1097/MD.0000000000000913

**Published:** 2015-06-12

**Authors:** Hong-yuan Shi, Xue-song Zhao, Fei Miao

**Affiliations:** From the Department of Radiology, Ruijin Hospital, Shanghai Jiaotong University School of Medicine, Shanghai, China (H-yS, X-sZ, FM).

## Abstract

The aim of this study is to identify the key computed tomography (CT) imaging findings and clinical characteristics of pancreatic metastases for its differential diagnosis. CT images and clinical features of 18 patients with 36 histopathologically proven pancreatic metastases were retrospectively reviewed. The primary malignancy included non-small cell lung cancer (NSCLC) (n = 7), gastrointestinal carcinoma (n = 5), renal cell carcinoma (RCC) (n = 3), osteosarcoma (n = 1), cardiac sarcomas (n = 1), and neuroendocrine ethmoid sinus carcinoma (n = 1). Pancreatic metastases were metachronous in 12 patients (ranging from 4 to 72 months). Tumor markers were elevated for 8 patients, of which 7 patients had NSCLC and gastrointestinal carcinoma, and 1 patient had osteosarcoma. Metastases from NSCLC and gastrointestinal carcinoma frequently presented as small well-circumscribed lesions, with homogeneous or rim enhancement, and or local pancreatic infiltration instead of focal mass, mimicking local pancreatitis. Neuroendocrine ethmoid sinus carcinoma affecting the pancreas also exhibited local pancreatic infiltration. Metastases from RCC and cardiac sarcomas had typical characteristics of hypervascular lesions. Osteosarcoma metastasizing to pancreas had special manifestation, that is, cystic lesion with thick wall and calcification.

Although pancreatic metastases have a broad spectrum of CT appearances, lesions from some types of primary tumors exhibited characteristic imaging features, which, in combination with oncological history, will contribute to correct diagnosis.

## INTRODUCTION

The majority of pancreatic carcinomas are primary, and among these, more than 90% are of ductal origin. Secondary tumors that metastasize to pancreas from remote sites constitute a quite small but an important category as it usually affects the treatment decision. Clinical data indicate that the incidence of pancreatic metastases varies from 2% to 5% of all pancreatic malignant tumors.^1^ However, with prolonged survival time partly due to the advancement of radiotherapy and chemotherapy, pancreatic metastases have been on rise over the past several years. Since most patients are asymptomatic or present with non-specific symptoms, the lesions are usually prone to be misdiagnosed or missed. In autopsy series, approximately one-third of them are clinically misdiagnosed as primary neoplasm, particularly if the interval is long.^2^

Because of the rarity of pancreatic metastases, more attention is required for radiologic diagnosis. Imaging, including ultrasound (US), endoscopic ultrasound-guided fine-needle aspiration (EUS-FNA) and CT, plays a prominent role in the diagnosis and management of pancreatic neoplasm. Although several studies have demonstrated the diagnostic value of EUS-FNA for accurate characterization of pancreatic metastases, it is invasive and depends on operators’ skill.^3,4^ On the other hand, CT is the most commonly used noninvasive modality for evaluation of oncologists during follow-up. To our knowledge, previous reports of the CT features for this tumor concentrated mainly on metastases from renal cell carcinoma (RCC).^1,5–8^ In order to increase the awareness of this entity among radiologists and clinicians, we undertake a review of the spectrum of CT findings and clinical features of pancreatic metastases in 18 patients, which will provide reference information for clinical treatment.

## PATIENTS AND METHODS

### Patients

The ethics committee at Ruijin Hospital approved this retrospective study. Written informed consent was obtained from all patients. From a computerized search of our hospital's pathology files and medical records from September 2000 to December 2014, we identified 18 patients with histopathologically proven pancreatic metastases who had undergone surgical treatment or cytological examination. The patients with malignancies arising from adjacent organs, lymphoma or metastatic lymph nodes that directly invade the pancreas were excluded. Available clinical data included gender, age, primary tumor, clinical symptoms, the interval from diagnosis of primary tumor to subsequent detection of metastases, and serum tumor markers.

### CT Scanning

Computed tomography scans were performed by using a 16-slice or 64-slice multidetector-row CT scanner (Lightspeed 16 or Lightspeed 64; GE Medical Systems Milwaukee, Wis). Images were reconstructed at slice thickness and interval ranging from 3 to 5 mm. Contrast enhanced CT was performed after the intravenous injection of iohexol (Omnipaque 300 or 350; Amersham, Shanghai, China) at an injection volume of 90 to 120 ml and an injection rate of 3 to 4 ml/s. As for the contrast enhanced CT, 7 patients underwent imaging both in the arterial phase and the portal venous phase; the other 11 patients underwent imaging only in the portal venous phase.

### Imaging Analysis

Computed tomography images were retrospectively evaluated in consensus by two experienced abdominal oncological radiologists who were blinded to clinical information and histological diagnosis. The following CT features were assessed: (1) location, (2) multiplicity (single or multiple), (3) size, (4) margin, (5) density, (6) enhancement pattern, (7) pancreatic duct dilatation (>2.5 mm) and common bile duct (CBD) dilatation (>7 mm), (8) vascular invasion, and (9) extrapancreatic metastases. The enhancement pattern was evaluated in two respects: first, the homogeneity (ie, homogeneous, heterogeneous, or rim) of the lesion enhancement was evaluated. Then, the relative degree of enhancement (ie, hyper-, iso-, or hypo-attenuating), compared with the enhancement of the surrounding pancreatic parenchyma, was evaluated in each phase of scanning.

## RESULTS

### Clinical Features

A total of 18 patients (males, 12; females, 6; mean age, 57.1 years; range, 38–76 years) with 36 pancreatic metastases were identified. The primary malignancy was NSCLC (adenocarcinoma) (n = 7), gastrointestinal carcinoma (n = 5), RCC (n = 3), osteosarcoma (n = 1), cardiac sarcomas (n = 1), and neuroendocrine ethmoid sinus carcinoma (n = 1) (Table 1). Six patients had simultaneous pancreatic metastases at the initial diagnosis of primary tumor, and 12 patients were found during follow-up. In the latter case, the mean interval was 36.6 months (6–72 months). Only 3 patients had clinical symptoms, which were nonspecific. Tumor markers were elevated in 8 patients, of which 7 patients had NSCLC and gastrointestinal carcinoma, and 1 patient had osteosarcoma.

**TABLE 1:**
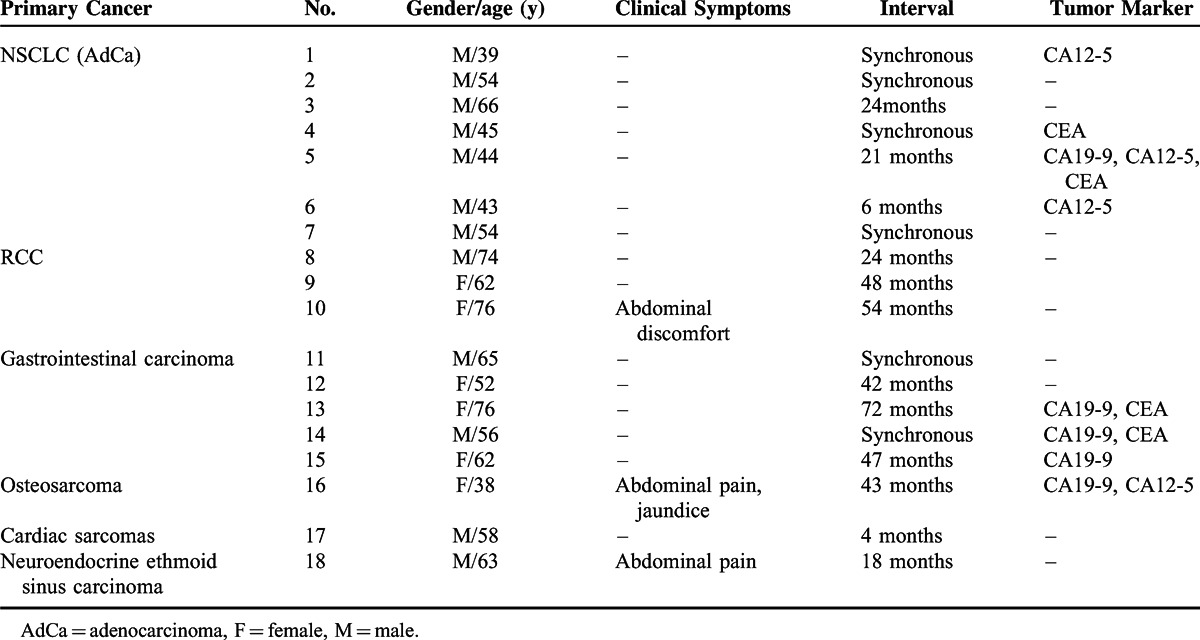
Clinical Data in 18 Patients with Pancreatic Metastases

### Imaging Characteristics

**FIGURE 1:**
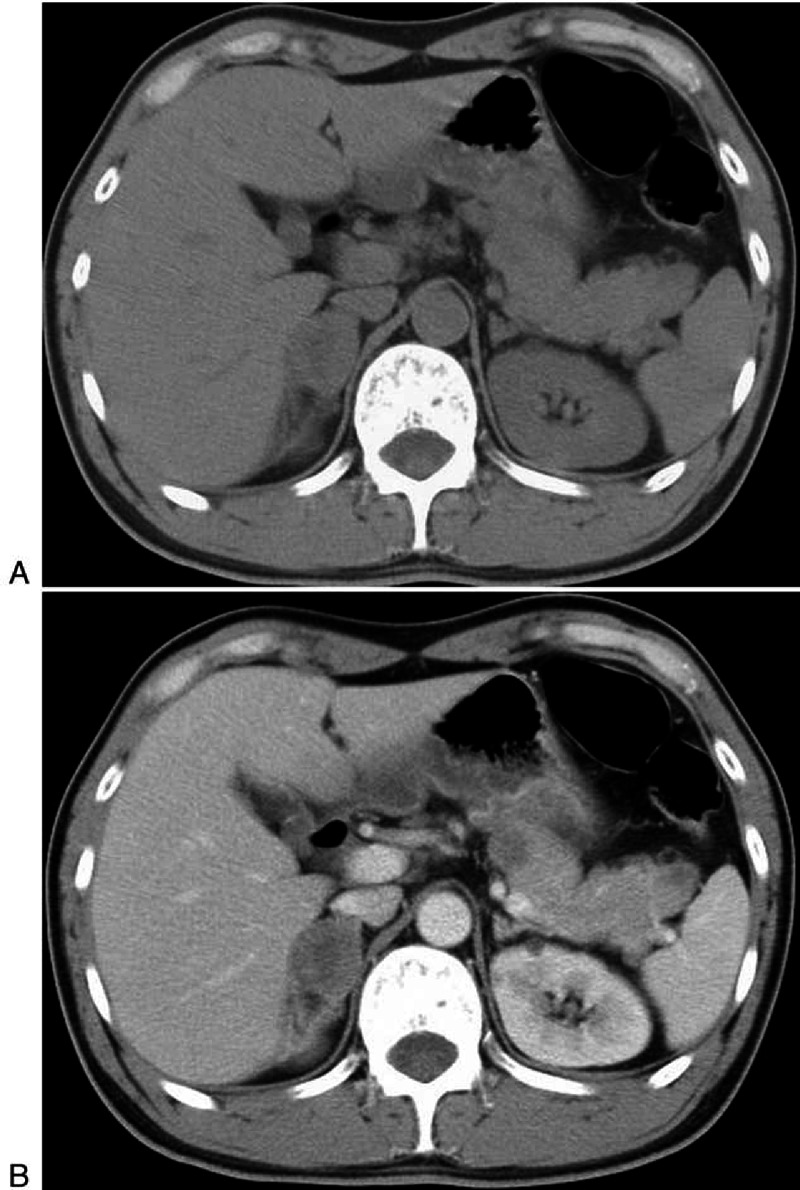
**A 39-year-old man with NSCLC (patient 1)**. A, On unenhanced CT the metastases in pancreas are very inconspicuous. B, Contrast enhanced CT show multiple hypo-attenuating lesions with homogeneous enhancement. Right adrenal metastasis is also seen.

**FIGURE 2:**
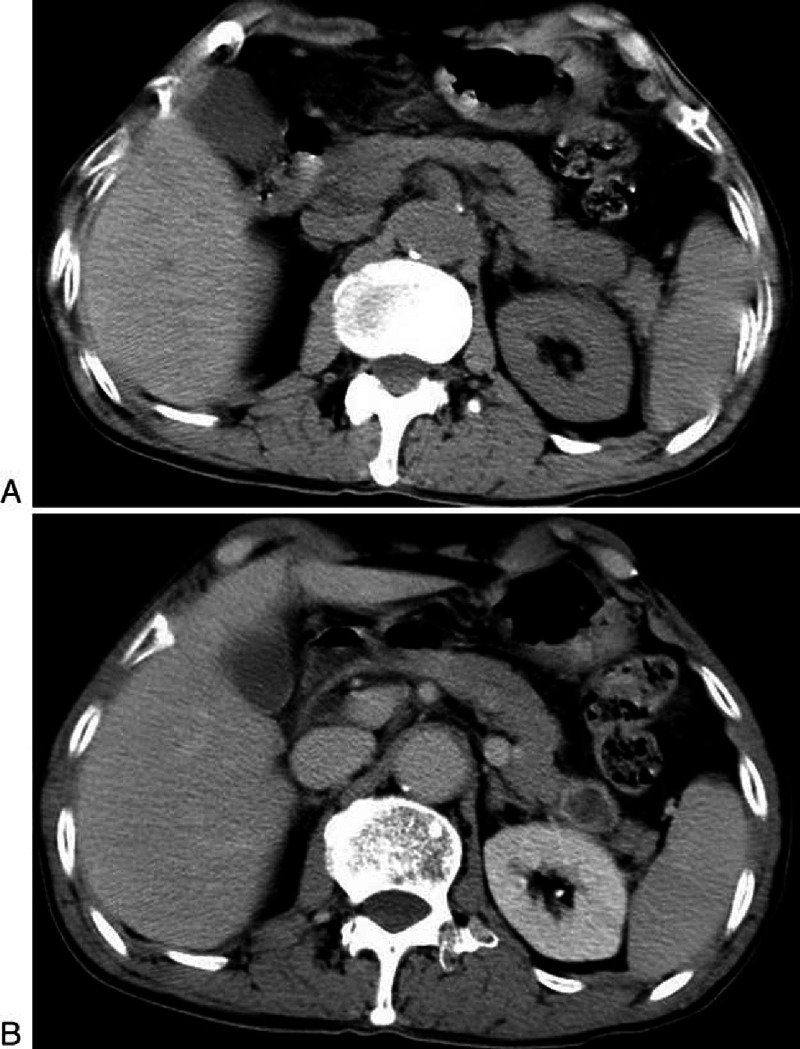
**A 66-year-old man with NSCLC (patient 3)**. A, On unenhanced CT the density of metastases in pancreas are nearly identical with normal pancreatic parenchyma. B, Contrast enhanced CT show the lesion in the tail had characteristic peripheral enhancement.

**FIGURE 3:**
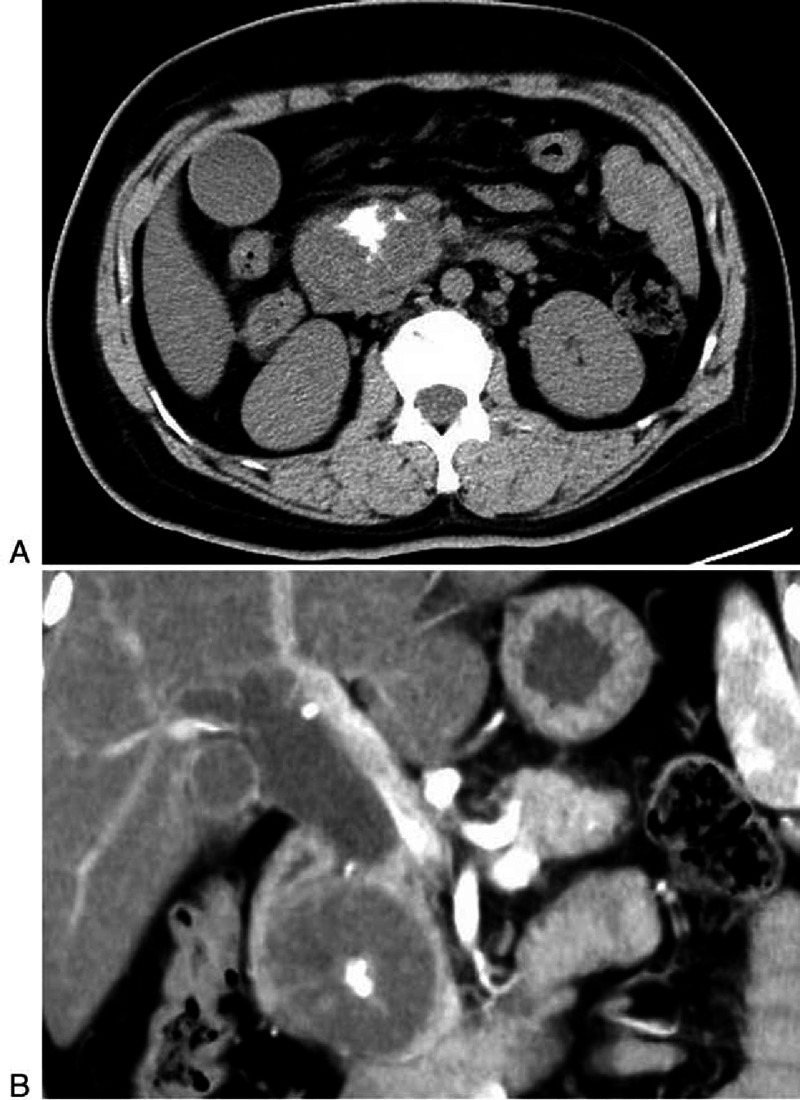
**A 38-year-old woman with osteosarcoma (patient 16)**. A, Unenhanced CT demonstrate the lesion in the pancreatic head presents cystic lesion, with calcification and partition inside the tumor. B, Coronal multiplanar reconstruction from contrast enhanced CT show evident dilation of common bile duct as a result of being oppressed by the well-defined mass.

By analyzing the image manifestations of 36 metastases in 18 patients, we determined the spectrum of CT features of pancreatic metastases (Table 2). The metastases were solitary in 12 patients, and multiple in 6 patients. Seventeen lesions were located in the pancreatic tail, 14 were in the body and 5 were in the head. The size ranged from 1.1 to 8.1 cm, and the largest lesion was from RCC. Most lesions (33/36) had a sharp margin, regardless of no true capsule. On unenhanced CT, 35 lesions were solid, presenting hypodense (18/35) or isodense (19/35) (Figures 1A and 2A). The remaining one from osteosarcoma presented a cystic mass with a peripheral enhancing thick cystic wall. An intratumoral calcification was also visible (Figure 3).

**TABLE 2:**
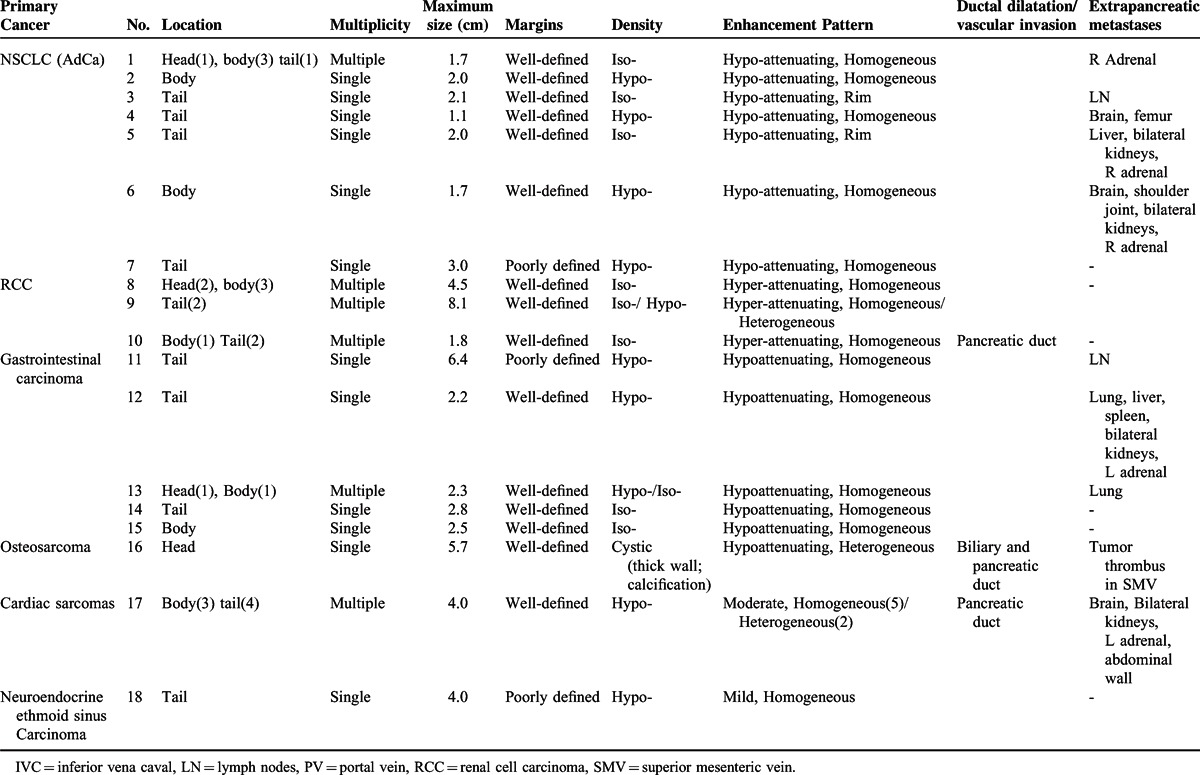
CT findings of 18 Patients with Pancreatic Metastases

**FIGURE 4:**
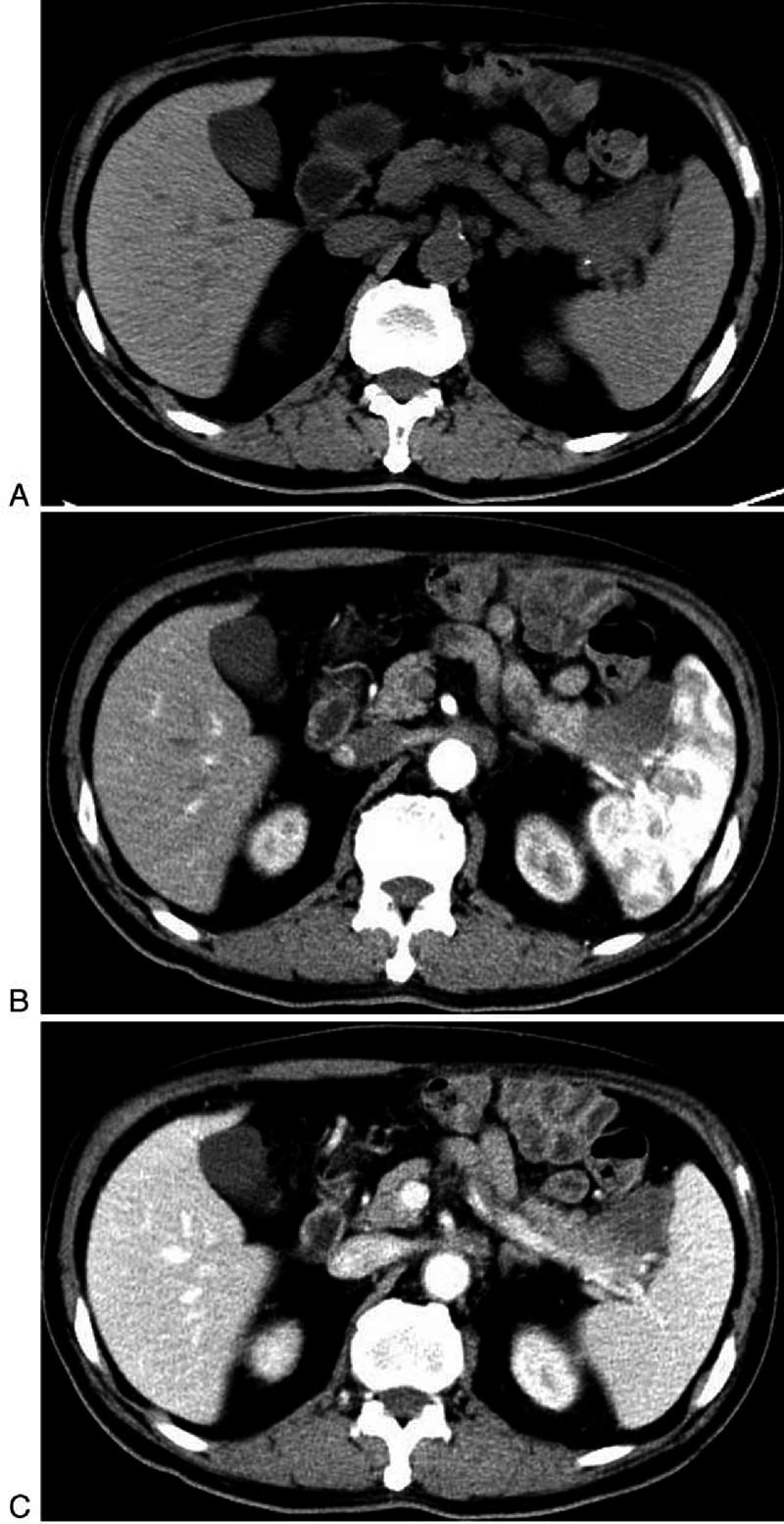
**A 63-year-old man with neuroendocrine ethmoid sinus carcinoma (patient 17)**. A, Unenhanced CT show a lesion in the pancreatic tail exhibite localized pancreatic infiltration instead of focal mass. B-C, Arterial phase and venous phase show mild enhancement of this metastasis.

To our attention and interest, metastases from NSCLC and gastrointestinal carcinoma had similar characteristics. The most common manifestation (7/12 patients, 12/17 lesions; 4 NSCLC and 4 gastrointestinal carcinoma) was hypodense or isodense mass with small size (1.1–2.8 cm in diameter) on unenhanced CT. In terms of enhancement pattern, they displayed hypoattenuated, compared to the normal enhanced pancreas, and homogeneous lesions, with well-circumscribed but lacked distinct intrapancreatic capsules (Figure 1B). The second presentation was local pancreatic infiltration with mild enhancement instead of focal mass in 2 patients (1 NSCLC and 1 gastrointestinal carcinoma). Besides, the lesion from neuroendocrine ethmoid sinus carcinoma exhibited this type of CT appearance (Figure 4). Metastases from NSCLC had a type of special appearance, that is, peripheral rim enhancement in 2 lesions (about 2 cm in diameter) (Figure 2B).

**FIGURE 5:**
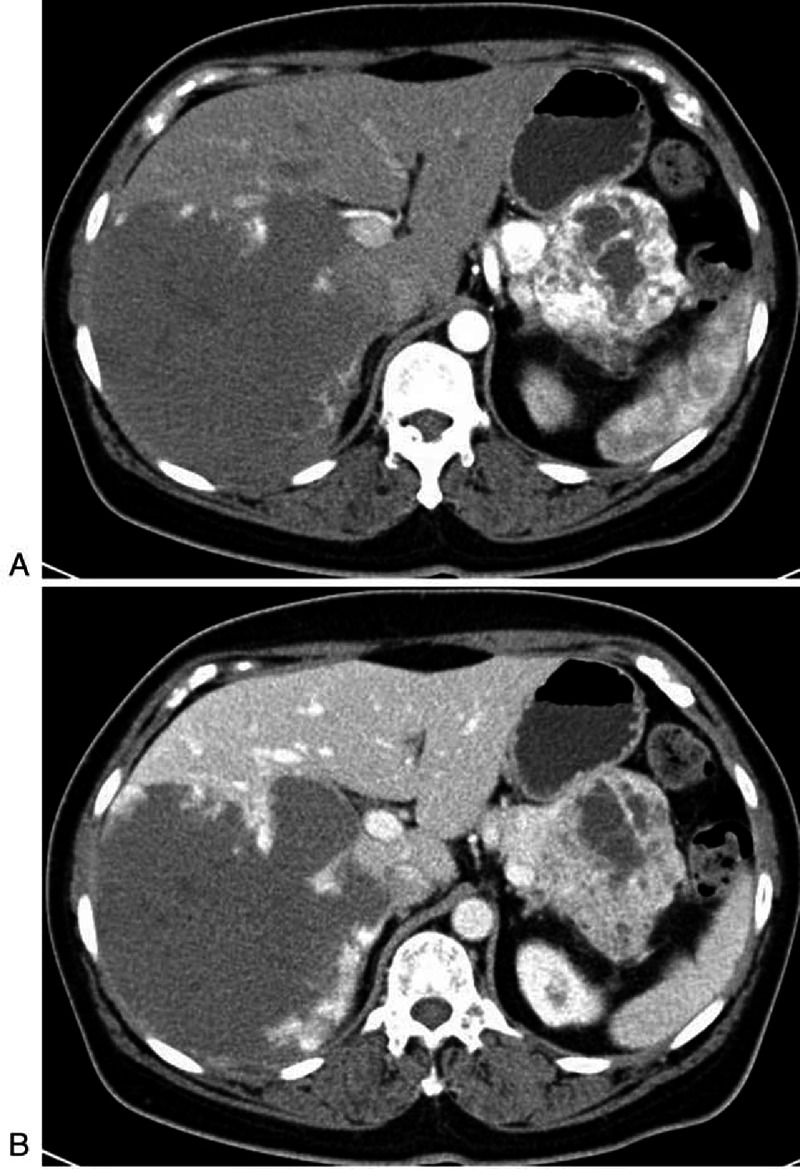
**A 62-year-old woman with RCC (patient 9)**. A, Arterial phase image from contrast-enhanced CT depict two hypervascular masses (close to together) in the pancreatic tail. The small one has homogeneous enhancement, while larger lesion has heterogeneous enhancement with many hypodense areas consistent with necrosis. B, The two masses wash out on the venous phase, similar to RCC. A giant hemangioma in the liver is also seen.

**FIGURE 6:**
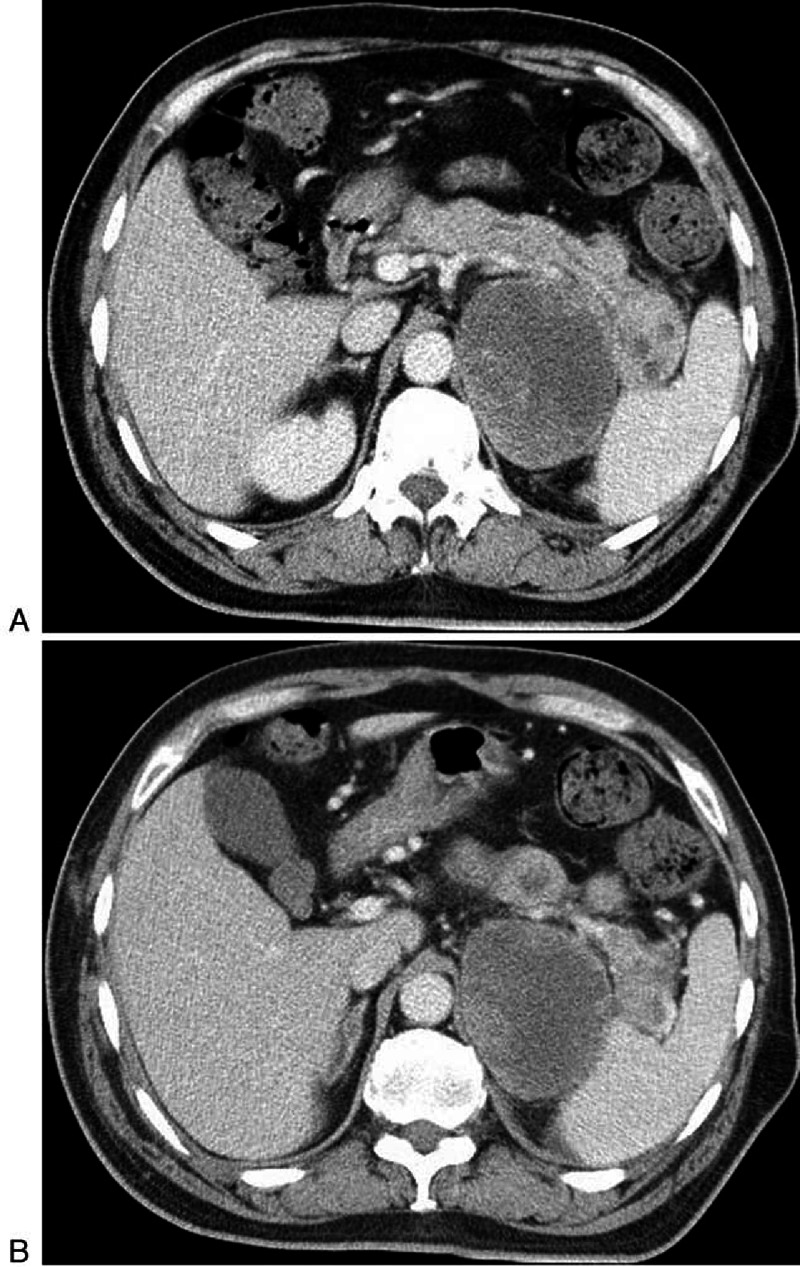
**A 58-year-old man with cardiac sarcomas with widespread metastases (patient 16)**. A-B, Contrast enhanced CT scans show that multiple well-defined and round lesions throughout pancreas with mild dilation of duct. Smaller lesions demonstrate moderate and homogeneous enhancement. Larger lesions show heterogeneous enhancement with internal hypodense areas consistent with necrosis.

Pancreatic metastases from RCC in 3 patients were all multiple and demonstrated obvious enhancement pattern, either homogeneously or heterogeneously, due to tumor necrosis (Figure 5). Two patients with RCC were followed up by CT examination after 11 and 13 months, respectively. The size of several lesions was further enlarged and the central areas had necrosis on follow-up CT scan. In addition, Cardiac sarcomas had the same features as RCC (Figure 6).

Pancreatic metastases involving the head region caused the dilation of CBD and the upstream main pancreatic duct, which resulted in the double duct sign in only one case (Figure 3; osteosarcoma). The upstream ductal dilatation of the main pancreatic duct was found in 2 cases (1 RCC and 1cardiac sarcomas). Distant metastases were found in 9 patients.

## DISCUSSION

In this retrospective study, we identified 18 patients with 36 pancreatic metastases, 7 from NSCLC, 5 from gastrointestinal carcinoma, 3 from RCC, 1 from osteosarcoma, 1 from cardiac sarcomas and 1 from neuroendocrine ethmoid sinus carcinoma. To our knowledge, this is the first report showing that cardiac sarcomas and neuroendocrine ethmoid sinus carcinoma affect the pancreas. The most common primary tumors reported in the literature included lung cancer, RCC, breast carcinoma, colorectal adenocarcinoma and melanoma,^9–12^ which did not differ substantially from our study.

The mechanism of the occurrence of metastases in an uncommon organ, such as pancreas, is poorly understood. According to previous studies, the reason may lie in the special biology of the tumor, such as some special molecular alterations and genetic associations. Or some tumor cells may have a high affinity for the parenchyma of the pancreas.^13^ Other studies hypothesize that modern chemotherapeutic protocols have somehow changed the pattern of biologic behavior and progression of oncological diseases. Though chemotherapy may indeed generate a partial or complete remission of malignancy, it generally brings another consequence of the changed biologically behavior of treated neoplasm.^1,13–15^

Analogous to primary neoplasm, the clinical symptoms of pancreatic metastases, including abdominal and back pain, nauseas and vomiting, anorexia, jaundice, gastrointestinal bleeding, if present, are variable and not specific.^16^ Serum carbohydrate antigen 19-9 (CA19-9) has a sensitivity of 81% and a specificity of 89% for the diagnosis of primary pancreatic adenocarcinoma.^17^ Especially, when the level of CA19-9 is increased progressively, its significance in the diagnosis of pancreatic adenocarcinoma becomes greater. In our series, elevation of CA19-9, carbohydrate antigen 12-5 (CA 12-5), and carcinoembryonic antigen were observed in 8 patients and most found in patients with NSCLC and gastrointestinal carcinoma. We deemed it was associated with the type and recurrence of primary tumors. However, we did not have continuous observation of the patients’ tumor markers since many patients were lost to follow-up. In addition, because of very few number of cases tested in our study and lack of researches in this area, further investigation of the significance of tumor markers in early diagnosis of pancreatic metastasis is required.

Currently, since improved accessibility to clinical surveillance or follow-up for primary tumors, imaging studies such as CT are more frequently performed in patients with oncology history.^18,19^ It is important to note that an appropriate imaging protocol is essential for the diagnosis of pancreatic metastases because most lesions (19/36) in our study demonstrated as an iso-attenuating tumor, with small size and without deformation of pancreatic contours, which may fail to be appreciated on unenhanced images. Based on our observation, enhanced CT examination should be favored as the most sensitive method for early detecting pancreatic metastases during follow-up for oncologists.

In our study, the most common primary tumors were NSCLC and gastrointestinal carcinoma. It was also interesting that metastases from NSCLC and gastrointestinal carcinoma in our case series showed similar CT imaging characteristics. We speculated that this might be related to the pathological type, since most of them were adenocarcinoma. They exhibited hypovascular lesions, analogous to pancreatic ductal adenocarcinoma (PDAC). However, unlike PDAC, most metastases tended to be well circumscribed although they lacked true capsules. Another unique feature was the absence of secondary changes caused by PDAC, such as dilatation of the upstream pancreatic duct or pancreatic parenchymal atrophy, which was consistent with previous studies on the imaging characteristics of metastases involving pancreas.^20,21^

Metastases from RCC have typical characteristic of hypervascular lesions.^1,6,7^ Moreover, some undifferentiated or aggressive neoplasm also tends to enhance perceptibly, such as cardiac sarcomas in our cases. It is worth noting that neuroendocrine carcinoma metastasizing to the pancreas is not only rare but also different from primary neuroendocrine carcinoma (NET). However, there was only one case in our series, which was also the first reported so far. Thus, further studies with more cases are needed. In addition, the CT features of metastases from osteosarcoma in our case were also peculiar. It manifested as a cystic tumor with thick cystic wall and calcification, which was difficult to distinguish from mucinous cystadenoma.

In terms of enhancement pattern, several studies demonstrated that some large lesions (>1.5 cm) usually manifested periphery enhancement with central low attenuation mainly due to necrosis, while smaller lesions tended to demonstrate homogeneous enhancement.^9,18,22^ In our series, two lesions with the maximum diameter of greater than 1.5 cm exhibited rim enhancement. However, we also observed tumors larger than 1.5 cm displayed homogeneous enhancement. Considering that the enhancement of tumors mainly depends on the blood supply to the masses instead of the size, we hypothesize that even though tumors grow large, cystic degeneration and necrosis may not occur as long as the blood supply is good, which is shown in our cases.

Differentiation of pancreatic metastases from primary neoplasm is an important unmet clinical need. The differential diagnosis of hypervascularized metastasis is large, but the primary consideration is NET. Other entities such as intrapancreatic accessory spleen, arteriovenous fistulas, or aneurysms of the splenic artery may occur but are unusual. In our clinical practice, we also find that a relatively small number of gastrointestinal stromal tumors can grow outward exophytically from the stomach or duodenum toward the pancreas, making it difficult to distinguish a lesion originating from pancreas. Moreover, these masses can show marked enhancement on arterial phase imaging and can become necrotic and cystic in appearance as they grow larger. These features can be very suggestive of a pancreatic NET. As a result, in some rare cases, this entity should be included in differential diagnosis when encountering hypervascularized lesions in the pancreatic head.

In diagnosing hypovascular metastases, the following must be considered: primary pancreatic carcinoma, lymphomas, and local pancreatitis. As shown in our study, three lesions, which exhibited localized pancreatic infiltration instead of focal mass, were misdiagnosed as local pancreatitis. Besides, an example of osteosarcoma metastasizing to the pancreas had an artificial pancreatic cystadenomas in the pancreatic head, presenting cystic mass with calcification and also causing evident dilatation of CBD and pancreatic duct in a young female patient, raising the differential features for pancreatic cystadenomas.

In conclusion, this study described comprehensive CT imaging features of pancreatic metastases. Although pancreatic metastases have a wide range of CT appearances, metastases from some kinds of primary tumors may have some typical imaging characteristics. Furthermore, the oncological history is extremely important and will contribute to correct diagnosis of pancreatic metastases.
